# Struma ovarii and peritoneal strumosis during pregnancy

**DOI:** 10.1186/s12884-021-03815-4

**Published:** 2021-05-02

**Authors:** Zheng Li, Jingxue Wang, Qian Chen

**Affiliations:** Department of Obstetrics and Gynecology, Peking University First Hospital, 100034 Beijing, P.R. China

## Abstract

**Background:**

Struma ovarii is a special type of ovarian dermoid cyst and accounts for approximately 2–3 % of all dermoid tumours. Benign struma ovarii may manifest as distant metastasis, called peritoneal strumosis, which makes it biologically similar to malignancy, and has been reported in limited cases but never discovered during pregnancy.

**Case presentation:**

We report a patient with a history of right struma ovarii cystectomy. During pregnancy, pelvic masses with non-specific clinical presentation were found again. During the caesarean section, contralateral struma ovarii with dissemination of nodules in the peritoneal cavity was found, and pathology revealed that the masses were thyroid follicle ovarian goitres.

**Discussion and conclusions:**

Recurrent benign struma ovarii with extraovarian dissemination is a rare aggressive clinical manifestation different from malignancy. It is emphasized that adequate assessment and complete resection of suspicious masses are of great importance.

## Background

Struma ovarii, in which thyroid tissue constitutes more than 50 % of the component, is a special type of ovarian dermoid cyst and comprises approximately 2–3 % of all dermoid tumours [1, 2]. It is mostly benign, with malignant transformation only occurring in 0.5–10 % of cases [4–9]. Benign cysts may manifest as distant metastases, which makes them biologically similar to malignant tumours. This phenomenon is also called peritoneal strumosis and has been reported in limited cases. Since the operations performed for benign and malignant tumours are quite different, preoperative assessments are of great importance. Struma ovarii is still rarely reported during pregnancy, and the adnexal mass is difficult to detect during that time [3].

We present a case of a woman with a history of cystectomy due to right struma ovarii 10 years prior. During this pregnancy, pelvic masses with nonspecific clinical presentation were found again with prenatal evaluations, including transvaginal ultrasonography and serum tumour biomarker assessment, which made it difficult to determine if they were benign or malignant. During the caesarean section, contralateral struma ovarii with dissemination of nodules in the peritoneal cavity was found, and pathology revealed that the masses were thyroid follicle ovarian goitres. We present a case of struma ovarii-complicated peritoneal strumosis, which has never been reported during pregnancy.

## Case presentation

The patient was a 39-year-old nulliparous woman who had a history of laparoscopy for bilateral ovarian cystectomy 10 years prior; the cyst wall was intact without spillage. The pathological examination showed that the left mass was serous cystadenoma, and the right mass was benign struma ovarii. She was then followed up with periodic ultrasonic examinations, which showed no evidence of structural disease during the 8-year follow-up. However, a routine ultrasound examination revealed an irregular multilocular cystic-solid mass (measuring 31 × 26 × 24 mm) with heterogeneous echogenicity in the left ovary region approximately 2 years ago.

### Investigations

**Fig. 1:**
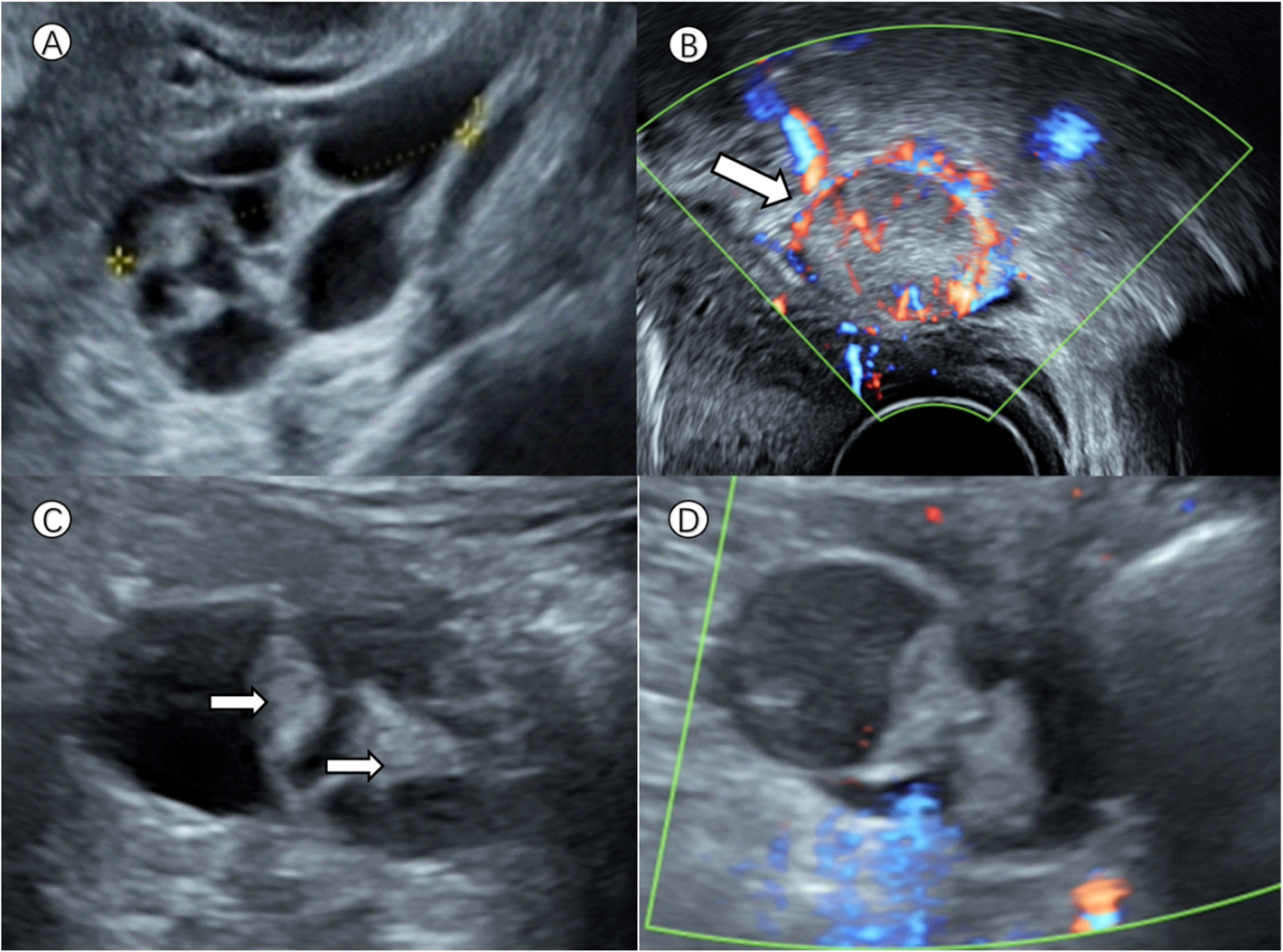
Greyscale and Doppler ultrasound images of the pelvic masses in a 39-year-old pregnant woman. **a** shows the period of the first trimester, and the cyst content was anechoic with thick septa. **b**, **c **and **d** were from the third trimester. **b** shows a solid hypoechoic area with rich blood flow signals in the Douglas pouch (white arrow). There were two papillary projections approximately 22 mm (arrows: **c**) with smooth contours and no vascularization on power Doppler (**d**)

The patient was admitted to our hospital at the 39th week of pregnancy for elective caesarean section due to a funnel-shaped pelvis. The obstetric condition was stable without pain or vaginal bleeding, and the thyroid function examination in early pregnancy showed no obvious abnormality. During the first trimester, ultrasound re-examination showed that the mass was approximately 39 × 37 × 26 mm with anechoic cyst fluid separated by thick septa, and a patchy irregular hypoechoic area (29 × 11 × 5 mm) between the cervix and the rectum was observed. In addition, a solid hypoechoic area whose diameter was approximately 22 mm with rich blood flow signals could be detected in the Douglas pouch(Figure1). Since there was not a very strong suspicion of malignancy and the clinical presentation was continuously uneventful, gynaecological experts recommended that surgery should be postponed until delivery, and rigorous monitoring should be performed unless there is a very clear indication, such as torsion or a very strong suspicion of malignancy. In the third trimester, ultrasound examination revealed that there were two papillary projections with smooth contours and moderate vascularization on the internal cyst wall of the left ovarian cyst. The other mass had no significant changes. The serum CA125 value gradually decreased from 124.7 U/mL in the first trimester to 25.91 U/mL in the third trimester, and the CA72-4 value increased from 14.77 U/mL in the second trimester to 21.14 U/mL in the third trimester; the level of human epididymis protein-4 (HE-4) was normal. The thyroid status was unknown during pregnancy since the diagnosis of struma ovarii was not suspected. Close periodical clinical and sonographic evaluations were performed to detect changes in the size and features of the masses.

#### Treatment

**Fig. 2:**
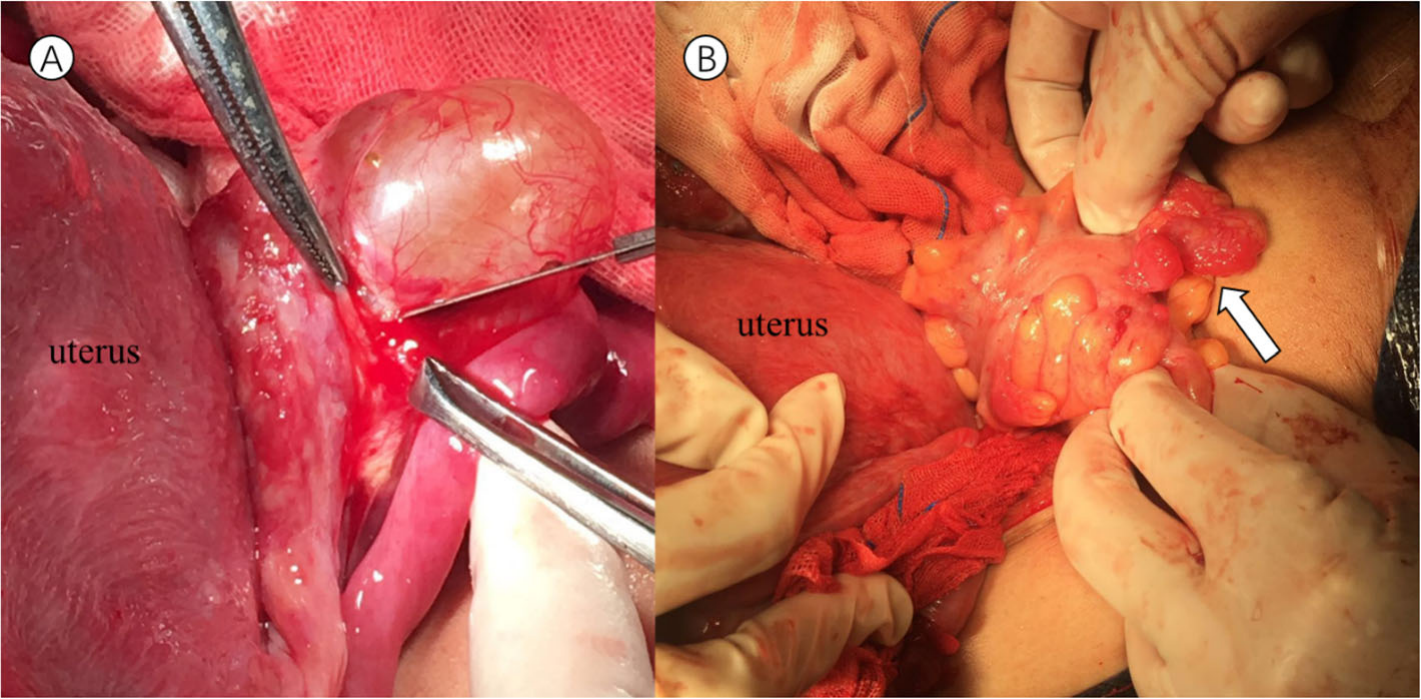
Pictures during the operation. **a** was the left ovarian cyst with a smooth wall. **b** shows the wall of the sigmoid colon with multiple nodules that presented with a smooth surface and gelatinous composition (white arrow)

**Fig. 3:**
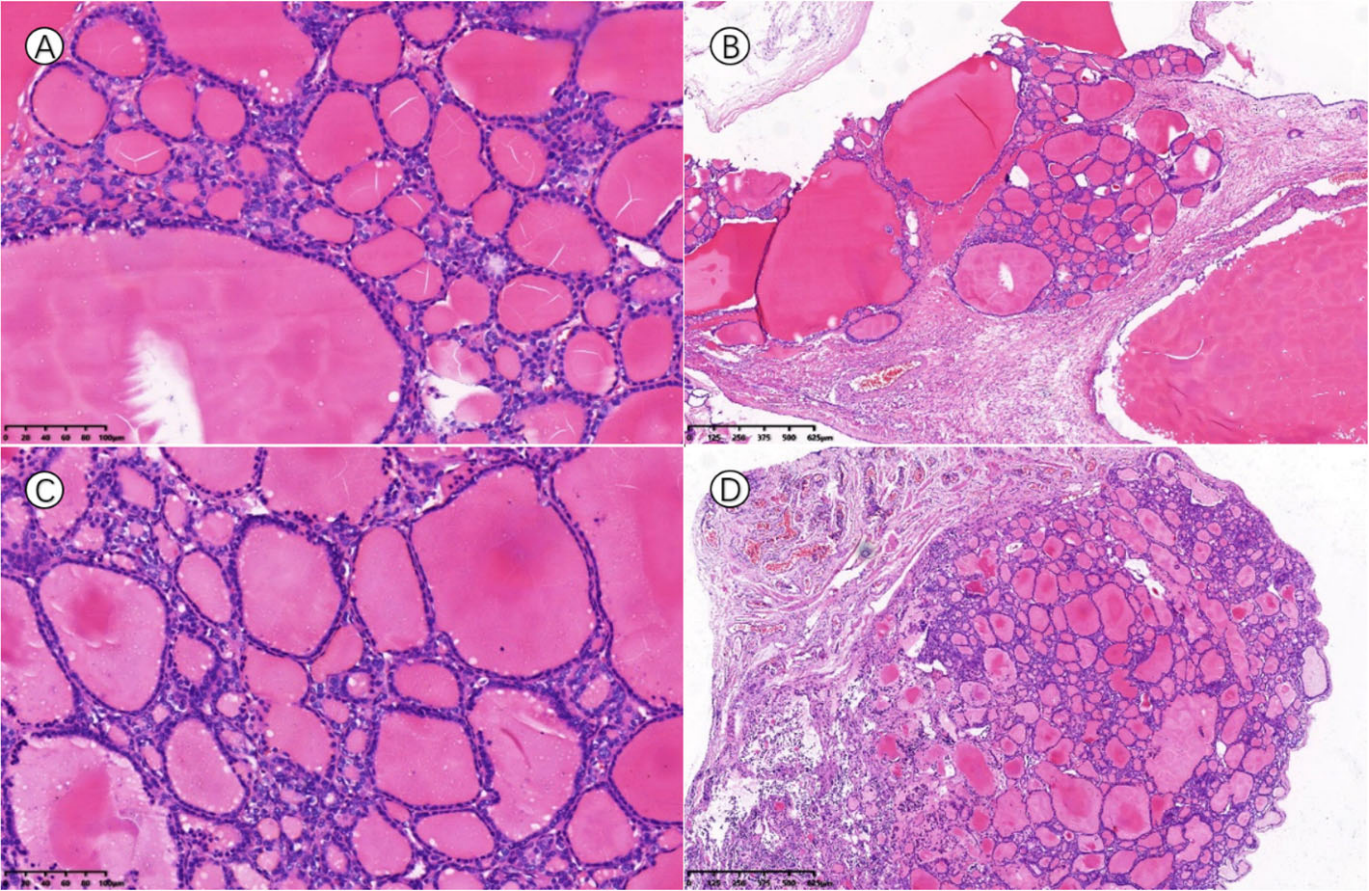
Pathology examination of resected ovarian mass and pelvic nodules. Haematoxylin-eosin staining showed thyroid follicles consistent with struma ovarii. **a** and **b** show pathology examination of the left ovarian mass. **c** and **d** were from the wall of the sigmoid colon and the surface of the uterus, respectively. The teratoma contained entirely thyroid tissue (**b** and **d**) composed of follicles filled with eosinophilic colloid material and lined by a single layer of cuboidal or columnar epithelial cells with uniform bland nuclei and eosinophilic cytoplasm (**a** and **c**)

After the delivery of a live, healthy female newborn weighing 2970 g through caesarean section, the pelvic organs were inspected thoroughly in a clockwise manner. There was no obvious ascites or fluid in the pouch of Douglas. Intraoperative exploration of the upper abdomen, including the liver, stomach, spleen, etc., and the contralateral ovary revealed a healthy appearance without gelatinous cysts. The left ovarian mass was multicystic and oval in shape. It was removed from the abdomen without any spillage of contents, and the cyst wall was smooth without papillary structures and separated by thick fibrous septa. In addition, multiple nodules that presented with smooth surfaces and gelatinous compositions were identified on her right fallopian tube, uterus, urinary bladder, pelvic wall and wall of the sigmoid colon. There were no macroscopically enlarged lymph nodes. Frozen section were then assessed because the extraovarian disseminated nodules were considered malignant (Fig. 2). In the pathological report, thyroid follicles of various sizes were observed in the excised tissues, presenting as nodular goitre changes. The operation was successful with resection of the whole visible mass and no lymph nodes. Five days later, permanent pathology reported that the mass of the left ovary consisted of densely packed monotonous thyroid follicles, morphologically mimicking follicular adenoma. The thyroid follicles had mild normal-appearing epithelial morphology and were consistent with struma ovarii (Fig. 3). Furthermore, the other part was considered to be the result of disseminated implantation of an ovarian goitre.

### Outcome and follow-up

The recovery of the patient was uneventful, and the patient was discharged from the hospital 6 days after the operation. Thyroid function tests performed postoperatively showed values within normal limits. It was also recommended that transvaginal ultrasound should be regularly performed to monitor the adnexal mass during the patient’s outpatient follow-up visits.

## Discussion and conclusion

This is a case of recurrent benign struma ovarii with extraovarian dissemination, which is a rare aggressive clinical manifestation. Struma ovarii accompanied by peritoneal or systemic dissemination but without malignant histological manifestations is termed peritoneal strumosis, which is controversial and problematic [10]. Its pathologic examination shows multiple nodules of mature thyroid tissue with features similar to those of struma ovarii. Most authors think that struma ovarii with extraovarian dissemination is highly differentiated follicular carcinoma (HDFCO) arising from benign struma ovarii and is evidence of malignancy [10]. HDFCO consists of benign-looking thyroid follicles but spreads beyond the ovary [11]. This neoplasm is considered biologically malignant due to its metastatic potential, which is a rare entity with only a few cases previously reported [12]. The relationship of HDFCO to peritoneal strumosis deserves further consideration because ovarian and peritoneal lesions are histologically indistinguishable, and some authors think peritoneal strumosis is a special type of HDFCO [10]. To the best of our knowledge, the cytopathological features of HDFCO have not been clearly described in the English literature [11].

It is difficult to predict the probability of metastasis and recurrence of struma ovarii, which may be related to the size of the goitre component [13, 14]. This patient was a pregnant female who had undergone ovarian cystectomy. An ovarian mass was found before pregnancy and reoccurred during the subsequent pregnancy, external ovarian masses were gradually revealed, and disseminated masses were discovered during caesarean section. Recurrence may be associated with the initial conservative surgery of cystectomy or oophorectomy alone to preserve fertility. Although masses were removed completely without macroscopic residuals, microscopic lesions may not be reliably ruled out. In addition, pregnancy has been speculated to be a favourable environment for the progression of struma ovarii, despite its rarity. Some epidemiologic studies have also shown that pregnancy may be associated with a transient increased risk of thyroid cancer, although the data are limited [15, 16]. This is based on the structural similarity between human chorionic gonadotropin (HCG) and thyroid-stimulating hormone (TSH). Thus, HCG can stimulate the TSH receptors of struma ovarii [17] and potentially promote tumour growth during pregnancy.

The metastasis and recurrence of struma ovarii prompt thorough evaluation and close surveillance after the initial conservative treatment. The typical approach combines serum tumour biomarkers, ultrasonography and computerized tomography (CT) or magnetic resonance imaging (MRI) scans. Tumour markers have significance in indicating malignant ovarian tumours [18], but they are inaccurate during pregnancy. Regular transvaginal ultrasound during pregnancy is the most frequent imaging examination and is a primary modality for the identification of any ovarian mass. A typical feature of struma ovarii is the presence of well vascularized solid tissue with a smooth margin that is vascularized in the Doppler study (“struma pearl”) [19]. MRI can be helpful at times due to its ability to distinguish between fluids and fat in diffusion‑weighted images, which may enhance the detection rate of malignancy. The classic MRI appearance of struma ovarii includes multiple intracystic-solid areas representing thyroid tissue, which show low‑signal intensity on T2‑weighted images and intermediate signal intensity on T1‑weighted images [20], but these characteristic features are not very easily interpreted on radiologic examination. Most cases are diagnosed on histopathology reports after surgery. Histologic appearances are often those of mature thyroid tissue, whereas areas resembling papillary hyperplasia, adenomatous goitre, adenomas or Hashimoto’s thyroiditis have also been reported. This highlights the importance of integrating clinical manifestations, laboratory examinations, imaging information and histopathology for the diagnosis and assessment of the metastasis and recurrence of struma ovarii. Although the diagnosis of ovarian masses in pregnancy is challenging, any persistent mass should be evaluated to exclude the risk of malignancy.

The effects of struma ovarii on the outcome of a normal pregnancy are rare. According to previous literature, there are no significant differences in prognosis between HDFCO and peritoneal strumosis. It is emphasized that adequate assessment and complete resection of suspicious masses are of great importance. Some articles reported that when the diagnosis of HDFCO was made, complete goitre removal and total thyroidectomy followed by radioiodine therapy (^131^I) should be performed and that serum thyroglobulin should be measured as a tumour marker for follow‑up [10], which is still controversial. At present, owing to the rarity and the lack of reliable prognostic factors, there is no consensus for the management of this condition after initial surgical diagnosis. All patients require indefinite follow-up, which should be based on pathologic and imaging parameters and individual characteristics.

## Data Availability

The datasets used and/or analysed during the current study are available from the corresponding author upon reasonable request.

## Abbreviations

HE-4: Human epididymis protein-4
HCG: Human chorionic gonadotropin
TSH: Thyroid-stimulating hormone
CT: Computerized tomography
MRI: Magnetic resonance imaging
HDFCO: Highly diff erentiated follicular carcinoma
